# Recurrent hemorrhagic pericardial effusion in a child due to diffuse lymphangiohemangiomatosis: a case report

**DOI:** 10.1186/1752-1947-4-62

**Published:** 2010-02-22

**Authors:** Shyam S Kothari, Sanjiv Sharma, Kinjal Bhatt, Ruma Ray, Sameer Bakhshi, Ujjwal Chowdhury

**Affiliations:** 1 Department of Cardiology, All-India Institute of Medical Sciences, Ansari Nagar, New Delhi, 110029, India; 2 Department of Cardiac Radiology, All-India Institute of Medical Sciences, Ansari Nagar, New Delhi, 110029, India; 3 Department of Pathology, All-India Institute of Medical Sciences, Ansari Nagar, New Delhi, 110029, India; 4 Pediatric Oncology, All-India Institute of Medical Sciences, Ansari Nagar, New Delhi, 110029, India; 5 Department of Cardiothoracic Surgery, All-India Institute of Medical Sciences, Ansari Nagar, New Delhi, 110029, India

## Abstract

**Introduction:**

Recurrent hemorrhagic pericardial effusion in children with no identifiable cause is a rare presentation.

**Case presentation:**

We report the case of a 4-year-old Indian girl who presented with recurrent hemorrhagic pericardial effusion. Diffuse lymphangiomatosis was suspected when associated pulmonary involvement, soft tissue mediastinal mass, and lytic bone lesions were found. Pericardiectomy and lung biopsy confirmed the diagnosis of diffuse lymphangiohemangiomatosis. Partial clinical improvement occurred with thalidomide and low-dose radiotherapy, but our patient died from progressive respiratory failure.

**Conclusion:**

Diffuse lymphangiohemangiomatosis should be considered in the differential diagnosis of hemorrhagic pericardial effusion of unclear cause.

## Introduction

Recurrent hemorrhagic pericardial effusion (HPE) is uncommon in children. Viral pericarditis, tuberculosis, neoplasm, connective tissue disease and drugs are typically responsible for HPE. We report a case of recurrent HPE that posed diagnostic and therapeutic challenges.

## Case presentation

A 4-year-old Indian girl was referred to us with a diagnosis of hemorrhagic pericardial effusion that recurred despite aspiration twice in the past 6 months. The child had insidious onset of breathlessness for six months and had episodes of lower respiratory tract infection. Pericardial effusion was detected on chest X-ray and hemorrhagic fluid was aspirated. She was started on antitubercular drugs with steroids, but her condition did not improve significantly. Our patient had normal development in her early infancy stage and normal growth prior to this illness. There was no family history of heart disease, developmental defects, tuberculosis or connective tissue disease.

**Figure 1:**
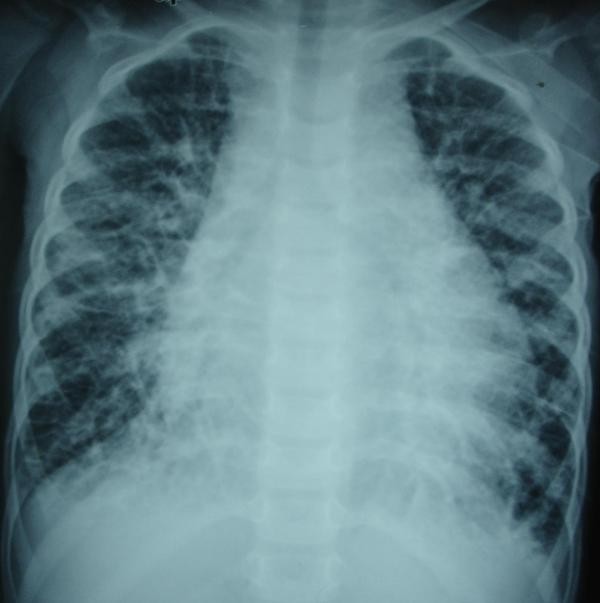
**Chest X-ray shows enlarged heart and increased markings in both lung fields**.

On examination, our patient was found to be in mild respiratory distress. She had a heart rate of 110/mt, BP of 90/60, respiratory rate of 30/mt, temperature of 37°C, and oxygen saturation of 96%. Her weight was 14 kg and her height was 110 cm. There were few basal crackles in her lungs and her heart sounds were distant. Chest X-ray showed marked cardiomegaly and streaky lung fields (Figure 1). Her hemoglobin count was 8.7 gm/dl, and her total leukocyte count (TLC) was 10600/mm^3 ^with 65% neutrophils. An echocardiogram showed large pericardial effusion (2.0 cm circumferentially) with evidence of tamponade. There was no structural lesion in her lungs. A total of 300 ml of hemorrhagic pericardial fluid was aspirated with a pigtail catheter in the pericardium. The pericardial fluid showed numerous red blood cells (RBCs) but no malignant cells were found. The adenosine deaminase in the fluid was not elevated. The bacterial and fungal cultures were sterile. Results of her abdominal ultrasound examination were normal.

**Figure 2:**
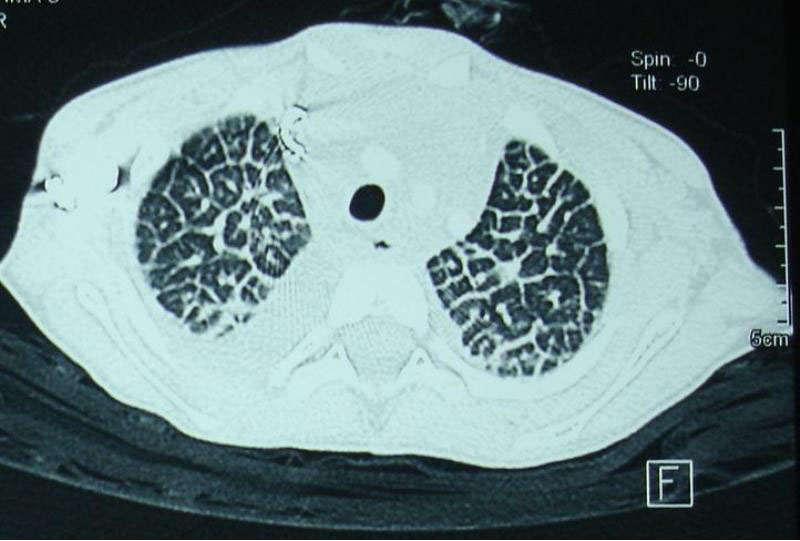
**High-resolution computed tomography image of the chest shows thickened interlobular septae with typical polygonal appearance of secondary pulmonary lobule**. Small amount of pleural fluid is seen.

The fluid in our patient's lungs re-accumulated within weeks of drainage. The antitubercular treatment and steroids were stopped. Meanwhile, results of her thyroid function tests were normal. Her rheumatoid factor, anti-nuclear antibodies, and antineutrophilic cytoplasmic antibodies were negative. She tested negative for human immunodeficiency virus (HIV) via rapid screening test. High-resolution computed tomography (HRCT) scan showed peculiar diffuse polygonal lobular architect (Figure 2) and soft tissue mediastinal mass. A needle biopsy of the mediastinal mass revealed only fat and connective tissues. Repeated pericardial fluid analyses for malignant cells were negative. Her platelet counts were 50 to 70,000/mm^3 ^on multiple occasions. She also tested negative for disseminated intravascular coagulation (DIC). Her bone marrow was normal.

**Figure 3:**
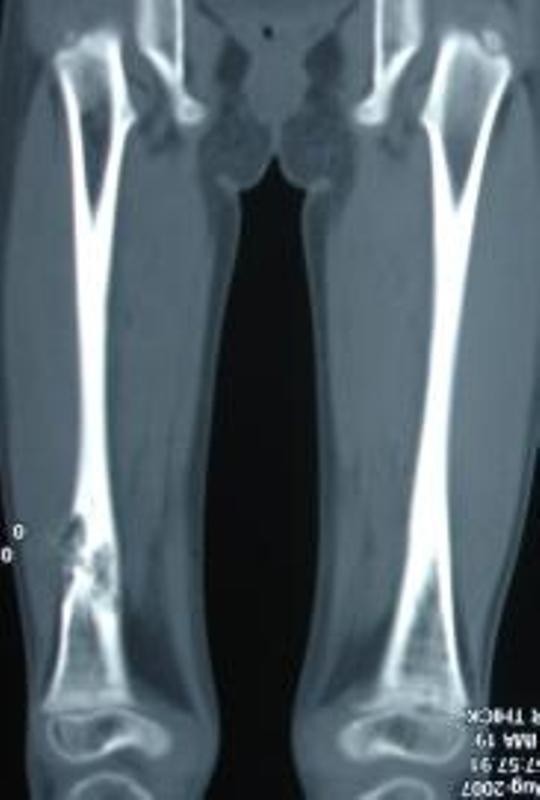
**A coronal computed tomography shows osteolytic lesion in the lower third of right femur**.

The diagnosis was unclear. A review of literature on similar HRCT picture [1] prompted a skeletal survey which showed lytic lesions in her bones (Figure 3). Consequently, diffuse multisystem involvement, lytic bone lesions and HRCT findings led to the diagnosis of diffuse lymphangiomatosis. The triglyceride levels in our patient's pericardial fluid were high, but her pericardial fluid was always hemorrhagic. During the course of her illness, she required multiple pericardiocentesis due to the large reaccumulation of fluid, as well as respiratory distress. Multiple blood transfusions were also given to our patient.

**Figure 4:**
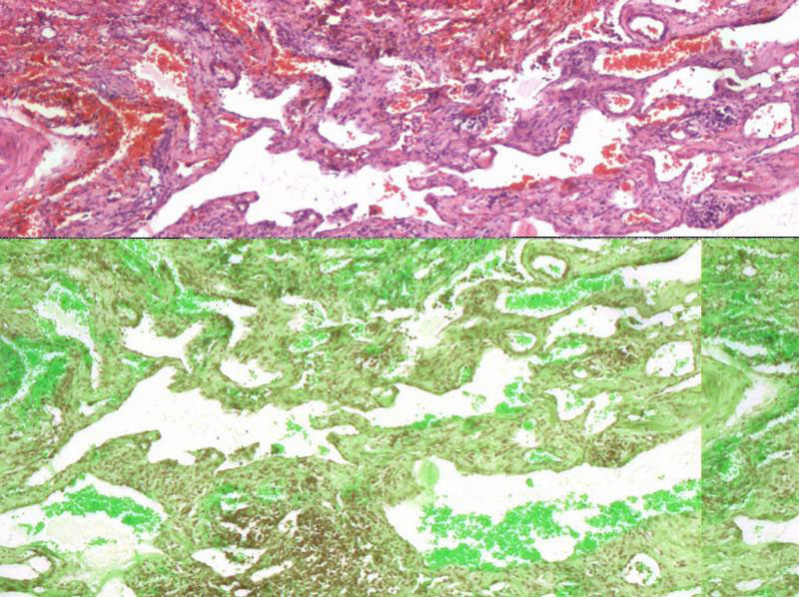
**Lung biopsy (hematoxylin and eosin imaging) shows multiple proliferating vascular spaces lined with endothelium infiltrating in the interstitium**. Lymphoid tissue is seen in the stroma. Some of the spaces contain blood.

Treatment with interferon alpha was discussed but her parents did not consent to it. Thalidomide (50 mg/d), octreotide and epsilon-aminocaproic acid were tried empirically, but her response to this treatment was not sustained. Low-dose radiotherapy of 20 Gy over 10 days were also given to her pericardium. A pericardiectomy was done after exhausting all options. Lung biopsy taken at that time showed diffuse hemangiolymphangiomatosis (Figure 4). There were numerous anastomotic proliferating, and cystic spaces in the pulmonary interstitium were lined by endothelial cells. The cells lining the spaces were CD31+, which is a marker of endothelial cells, although it does not differentiate vascular from lymphatic capillaries. Many of her capillaries contained blood. The connective tissue stroma was predominantly lymphoid. Our patient's pericardium also showed similar findings. A diagnosis of diffuse lymphangiohemangiomatosis was thus made. Our patient had progressive respiratory failure and died after two months.

## Discussion

Diffuse lymphangiomatosis is a rare, non-malignant but locally infiltrative multisystem disease that may involve any tissue except the brain [2]. The thorax, bones, and spleen are typically involved. The clinical course is highly variable, but disease occurrence and lung involvement in children suggest a poorer prognosis. To the best of our knowledge, lymphangiohemangioma presenting with recurrent hemorrhagic pericardial effusion has not been reported previously. The diagnosis in our case was delayed until we were able to recognize the multisystem nature of our patient's disease. A chylous pericardial effusion might have led to an earlier diagnosis, but the hemorrhagic effusion resulted from the hemangiomatous component of the angioma in the pericardium.

The histologic differentiation of lymphangiomatosis from hemangiomatosis is not always easy and the total picture favoured the label of lymphangiohemangiomatosis. In retrospect, this is not altogether surprising as the lymphatic endothelium does have embryological origin from the vascular system [2,3]. Similar mixed angiomatous patterns have been uncommonly recognized previously [4]. The specific marker for lymphatic endothelium Lyve-1, a lymphatic vascular endothelial receptor for hyaluronan, was not tested in our patient but has been found also in vascular tumors like Kaposi's sarcoma [5]. Likewise, it has not been tested in malformations like this one for a discriminatory value.

The terminology and nosologic relationship of soft tissue tumours of the lymphatic system can be confusing. Based on histopathological and clinical features, some authors suggest a classification into lymphangioma, lymphangiectasia, diffuse lymphangiomatosis, lymphatic dysplasia syndrome, and a host of other miscellaneous disorders [2]. Lymphangioma are focal proliferation of well-differentiated lymphatic tissues that can be cystic as in cystic hygroma. Primary lymphangiectasia presents in neonates and are characterized by dilated lymphatic channels that are remnants of otherwise normal fetal lymphatics. In lymphangiomatosis there is multifocal and more complex proliferation of mature lymphatic capillaries. The proliferating lymphatics are infiltrative. The dilated lymphatic channels may be of capillary size to several centimeters and is surrounded by connective tissue stroma containing lymphoid tissues and smooth muscle cells. The surrounding tissues are infiltrated but not destroyed.

Meanwhile, another cystic disease of the lungs, lymphangioleiomyomatosis, is an entirely different disorder that is seen in premenopausal women where the proliferating tissue destroys the surrounding lung tissues. The cells in this disorder are estrogen-positive smooth muscle cells [6].

Imaging with CT and magnetic resonance imaging (MRI) may provide diagnostic information typical of diffuse lymphangiomatosis [1]. The lytic lesions in the bones have sharp margins without periosteal reaction. Langerhans cell histiocytosis needs to be excluded in children with multiple organ involvement and lytic lesions in the bones. The thoracic involvement in diffuse lymphangiomatosis may produce soft tissue mediastinal mass, interstitial nodular lesions, and pericardial and pleural involvement, as in our patient. The lesions are isointense to muscle on T1-weighted images and hyperintense to fat on T2-weighted images on MRI, These do not show strong contrast enhancement. Whole body MRI with short tau inversion recovery sequence is considered a better method for delineating the extent of the involvement of diffuse lymphangiomatosis [1].

The etiology of lymphangiomatosis remains unclear. Recently, a number of growth factors that enhances lymphogenesis have been identified, including VEGF-a, VEGF-C, PDGF-BB, and angiopoeitin [6]. These findings may have a therapeutic role. In fact, the treatment of lymphangiomatosis with antiproliferative and anti-angiogenetic drugs like interferon alpha 2a and 2b have also been reported [7]. Long-term treatment for up to 30 months has been used, although the treatment is not always effective. Low-dose radiation has been effective in some cases [8]. Thalidomide has been tried for its anti-VEGF properties [9]. In the future, a better understanding of the molecular mechanisms of proliferation and/or triggers may lead to better outcomes.

## Conclusion

In conclusion, diffuse lymphangiohemangiomatosis may present with hemorrhagic pericardial effusion. A wider appreciation of this multisystem disease is warranted.

## Abbreviations

HPE: hemorrhagic pericardial effusion; HRCT: high-resolution computed tomography; MRI: magnetic resonance imaging; PDGF: platelet derived growth factors; VEGF: vascular endothelial growth factor.

## Consent

Written informed consent was obtained from our patient's parents for publication of this case report and any accompanying images. A copy of the written consent is available for review by the Editor-in-Chief of this journal.

## Competing interests

The authors declare that they have no competing interests.

## Authors' contributions

SK researched the literature and wrote the manuscript. SS analysed the imaging data. RR reported the slides and contributed to the discussions. KB researched the literature and helped in writing the manuscript. SB contributed intellectually to the correct diagnosis and suggested the novel therapy. UC performed the pericardiectomy. All authors read and approved the final manuscript.
